# Gene Editing of HIV-1 Co-receptors to Prevent and/or Cure Virus Infection

**DOI:** 10.3389/fmicb.2018.02940

**Published:** 2018-12-17

**Authors:** Alexander G. Allen, Cheng-Han Chung, Andrew Atkins, Will Dampier, Kamel Khalili, Michael R. Nonnemacher, Brian Wigdahl

**Affiliations:** ^1^Department of Microbiology and Immunology, Drexel University College of Medicine, Philadelphia, PA, United States; ^2^Center for Molecular Virology and Translational Neuroscience, Institute for Molecular Medicine and Infectious Disease, Drexel University College of Medicine, Philadelphia, PA, United States; ^3^School of Biomedical Engineering and Health Systems, Drexel University, Philadelphia, PA, United States; ^4^Department of Neuroscience, Center for Neurovirology, and Comprehensive NeuroAIDS Center, Lewis Katz School of Medicine, Temple University, Philadelphia, PA, United States; ^5^Center for Translational AIDS Research, Lewis Katz School of Medicine, Temple University, Philadelphia, PA, United States; ^6^Sidney Kimmel Cancer Center, Thomas Jefferson University, Philadelphia, PA, United States

**Keywords:** CRISPR/Cas9, HIV-1, CXCR4, CCR5, CD4

## Abstract

Antiretroviral therapy has prolonged the lives of people living with human immunodeficiency virus type 1 (HIV-1), transforming the disease into one that can be controlled with lifelong therapy. The search for an HIV-1 vaccine has plagued researchers for more than three decades with little to no success from clinical trials. Due to these failures, scientists have turned to alternative methods to develop next generation therapeutics that could allow patients to live with HIV-1 without the need for daily medication. One method that has been proposed has involved the use of a number of powerful gene editing tools; Zinc Finger Nucleases (ZFN), Transcription Activator–like effector nucleases (TALENs), and Clustered Regularly Interspaced Short Palindromic Repeats (CRISPR)/Cas9 to edit the co-receptors (CCR5 or CXCR4) required for HIV-1 to infect susceptible target cells efficiently. Initial safety studies in patients have shown that editing the CCR5 locus is safe. More in depth *in vitro* studies have shown that editing the CCR5 locus was able to inhibit infection from CCR5-utilizing virus, but CXCR4-utilizing virus was still able to infect cells. Additional research efforts were then aimed at editing the CXCR4 locus, but this came with other safety concerns. However, *in vitro* studies have since confirmed that CXCR4 can be edited without killing cells and can confer resistance to CXCR4-utilizing HIV-1. Utilizing these powerful new gene editing technologies in concert could confer cellular resistance to HIV-1. While the CD4, CCR5, CXCR4 axis for cell-free infection has been the most studied, there are a plethora of reports suggesting that the cell-to-cell transmission of HIV-1 is significantly more efficient. These reports also indicated that while broadly neutralizing antibodies are well suited with respect to blocking cell-free infection, cell-to-cell transmission remains refractile to this approach. In addition to stopping cell-free infection, gene editing of the HIV-1 co-receptors could block cell-to-cell transmission. This review aims to summarize what has been shown with regard to editing the co-receptors needed for HIV-1 entry and how they could impact the future of HIV-1 therapeutic and prevention strategies.

## Introduction

Human immunodeficiency virus type 1 (HIV-1) currently affects more than 37 million people worldwide with approximately 2 million people that are newly infected every year (Sengupta and Siliciano, 2018). Antiretroviral therapy (ART) has been shown to inhibit active viral replication, driving viral loads to undetectable levels (Maartens et al., 2014). While ART has transformed the clinical management of HIV-1 disease, it has not led to a cure of this disease due to the development of a residual level of virus often referred to as the latent reservoir (Huang et al., 2018). In an attempt to prevent or more recently treat infection entirely, a vaccine has been in development for more than three decades. While there have been numerous HIV-1 vaccine clinical trials, there has yet to be one that demonstrated significant clinical success. The only clinical trial to demonstrate some level of success was the RV144 trial (Rerks-Ngarm et al., 2009). Due to continued difficulties in continuing traditional vaccine strategies, researchers have turned to a number of alternative methods including gene editing to achieve a functional or sterilizing cure.

HIV-1 infects cells that express CD4 and the co-receptors CCR5 or CXCR4 (Maartens et al., 2014). The CD4, CCR5, CXCR4 axis is considered the classical route of HIV-1 entry, although there is a body of literature that demonstrates HIV-1 employs a number of methods to get into target cells (Kunsch et al., 1989). While CD4+ T cells represent the primary target of HIV-1, macrophages are also readily infected by HIV-1 (Arainga et al., 2017). It has been shown that HIV-1 can infect macrophages in a CD4 independent manner, leading to endocytosis of the virus (Harouse et al., 1989; Gobeil et al., 2012). In addition, there have been studies that have revealed a CD4 and CCR5 or CXCR4 independent method for HIV-1 infection of macrophages (Gobeil et al., 2012). This mechanism of entry has been largely attributed to the phagocytic nature of macrophages. While it is rare to have dendritic cells (DCs) infected by HIV-1, it is well established that HIV-1 can bind and stay bound to a DC receptor known as DC-SIGN (McDonald et al., 2003). In addition, it has been shown that CD169 plays a significant role in mediating HIV-1 capture by DCs. As HIV-1 buds from an infected cell it incorporates a glycosphingolipid (GSL) with a terminal α2,3 sialic acid residue known as GS3. GS3 is then able to bind to CD169 which has been shown to be upregulated in the presence of interferon (IFN) thereby contributing to cell-to-cell transmission (Gummuluru et al., 2014). These interactions allow DCs that have HIV-1 bound on the surface to interact with uninfected T cells, leading to an enhancement of infection, through cell-to-cell transmission.

To date, one patient, “the Berlin patient,” is considered cured of HIV-1. This patient received a bone marrow transplant from a donor who was homozygous for a CCR5 mutation known as CCR5Δ32. While the exact mechanism of how this patient defeated HIV-1 is still under investigation, it is largely believed that the CCR5 mutation was a key factor (Yukl et al., 2013). Although, it should be noted that this patient did have CXCR4-utilizing virus but surprisingly a rebound of this virus has not been observed. An additional transplant study was able to deep sequence the provirus from an HIV-1-infected patient. The gp120 V3 region from this patient was cloned into an HIV-1 backbone and it was shown that this virus was able to infect PBMCs through CXCR4 (Verheyen et al., 2018). After allogenic transplantation from a CCR5Δ32 donor, a CXCR4 utilizing virus rebounded in that patient. This has spawned research efforts to utilize gene editing technologies to manually reconstruct the CCR5Δ32 mutation (Tebas et al., 2014). Ideally this research would be aimed at either editing hematopoietic stem cells to give rise to cells that are naturally resistant to HIV-1 infection or edit peripheral blood mononuclear cells (PBMCs) and infuse them back into the patient.

**FIGURE 1:**
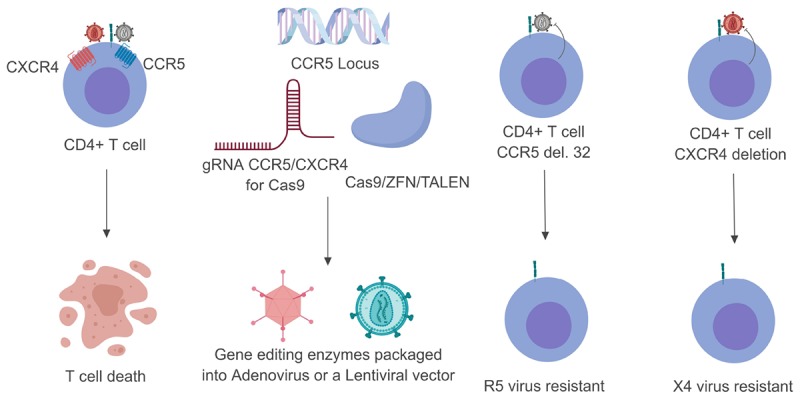
Schematic breakdown of gene editing strategies targeting CCR5 or CXCR4. Once the HIV-1 glycoprotein gp120 has made contact with CD4 it undergoes a series of conformational changes that allow it to bind to one of two co-receptors (CCR5 or CXCR4). This normally leads to T-cell death within 24– 48 h (left). By customizing either the Cas9 or ZFN system these endonuclease enzymes can be packaged into viral vectors and transduced into their target cells (middle). Upon successful genome editing these target cells can be rendered resistant to either CCR5- or CXCR4-utilizing virus (right). Gray viral particles indicate CCR5-utilizing virus (R5) while the red viral particles indicate an CXCR4-utilizing virus (X4).

Three main gene editing technologies have been used to edit CCR5 and CXCR4. These include zinc finger nucleases (ZFN), transcription activator–like effector nucleases (TALENs), and the clustered regularly interspaced short palindromic repeats (CRISPR)/Cas9 system (Wilen et al., 2011; Xu et al., 2017; Yu S. et al., 2018; Figure 1). While these systems aim to edit the genome, they go about it in two very different ways. ZFNs are made up of protein modules that recognize their target DNA sequence, as previously reviewed (Jabalameli et al., 2015). Attached to these sequence modules are Fok1 endonucleases which are responsible for catalyzing the double stranded break (DSB) in the target DNA (Zhu et al., 2013). TALENs act very similarly to ZFNs by having amino acid repeats that are capable of binding DNA and utilizing Fok1 as the nuclease effector (Joung and Sander, 2013). The CRISPR/Cas9 system works by utilizing a custom designed guide RNA (gRNA) sequence that complexes with the Cas9 endonuclease. The gRNA is broken into two main components. The protospacer is a 20 base pair (bp) sequence that binds to target DNA with sequence complementarity. The scaffold component has been shown to allow the gRNA to bind with the Cas9 endonuclease. Once the gRNA and the Cas9 have bound together to form a ribonucleoprotein complex, the Cas9 enzyme binds to a protospacer adjacent motif (PAM), normally an NGG sequence for Streptococcus pyogenes Cas9. Once the Cas9 has bound, the gRNA is able to bind with complementarity to its target site and a DSB will occur (Doudna and Charpentier, 2014).

There have been numerous studies performed with ZFN to edit the CCR5 gene in an attempt to stop HIV-1 infection. *In vitro* studies have shown that editing the CCR5 locus limits the number of cells HIV-1 can infect (Wang et al., 2014, 2017; Liu et al., 2017). Moreover, there have been a limited number of *in vivo* studies using ZFN to edit CCR5 (Wilen et al., 2011; Yi et al., 2014). These studies were able to show that even with successful gene editing HIV-1 was able to replicate, albeit to a lesser extent. While editing CCR5 confers resistance to CCR5-utilizing viruses, it doesn’t confer resistance to CXCR4-utilizing viruses. These results have led to a number of studies aimed at editing CXCR4. Preliminary results have shown that editing CXCR4 conferred resistance to X4 virus with minimal cytotoxicity (Hou et al., 2015; Yu S. et al., 2018).

Editing studies targeting CCR5 and CXCR4 have brought to light the problem of gene editing efficiency. This efficiency problem is highlighted in studies, utilizing humanized mouse models. These studies have shown that HIV-1 was able to replicate at the early time points but replication declines over time when compared to the untreated control. It is now believed that HIV-1 will replicate in cells that were not successfully modified and when those target cells decrease in number with time, there will be a simultaneous expansion in the number of edited cells ultimately limiting the infection (Xu et al., 2017). Data supporting this model of conferred resistance has been observed using CRISPR, ZFN, and TALEN therapeutic approaches. These gene editing technologies have been shown to successfully edit both CCR5 and CXCR4 in a population of cells. While these results are promising, an increase in gene editing efficiency for both co-receptors and enhancements to existing delivery systems will be necessary for these therapeutic approaches to be successful. In this review, we examine studies that have utilized different gene editing technologies to edit CCR5 or CXCR4 and discuss how different mechanisms of HIV-1 infection can be inhibited by editing the co-receptors needed for HIV-1 infection.

## Cellular Components That Are Involved in HIV-1 Entry Are Potential Targets to Stop Infection

To date, the process of HIV-1 entry has been dissected into three major steps: (1) HIV-1 gp120 recognizes host receptor CD4 followed by a conformational change of gp120 (Maddon et al., 1986; Sattentau and Moore, 1991; Kwong et al., 1998). (2) The restructured gp120 is able to recognize host co-receptor CXCR4 (Oberlin et al., 1996) or CCR5 (Alkhatib et al., 1996; Choe et al., 1996; Deng et al., 1996; Doranz et al., 1996; Dragic et al., 1996; Feng et al., 1996), which gives rise to the exposure of the hydrophobic fusion peptide on HIV-1, referred to as gp41. (3) The formation of a six-helix bundle using three gp41 subunits brings the plasma membrane and HIV-1 Env in close proximity, completing the membrane fusion event (Chan et al., 1997; Weissenhorn et al., 1997; Furuta et al., 1998; Markosyan et al., 2003). Intervention in any step of the HIV-1 entry process may establish an effective barrier to prevent new infections (Catalone et al., 2004; Thakkar et al., 2009; Passic et al., 2010). Indeed, research using different strategies to inhibit all three steps of the HIV-1 entry cycle have achieved resounding success. CD4 or CD8 molecules have been genetically engineered and chimerically coupled with the zeta-chain of the T-cell receptor; and as constructed, the expression of chimeric CD4 receptor molecules upon the recognition of HIV-1 Env would activate the effector function of these T cells and stop the new infection (Roberts et al., 1994; Yang et al., 1997). This strategy has led to two clinical trials that resulted in minimal impact on CD4 count and viral load in all patients examined in the study; however, a significant reduction of the HIV-1 reservoir was demonstrated in CD4 zeta-modified autologous CD4 and CD8 T cells (Mitsuyasu et al., 2000; Walker et al., 2000; Deeks et al., 2002). The possibility of interrupting the formation of the six-helix bundle during membrane fusion has also been explored as well. Research focused on peptide inhibitors has led to the development of enfuvirtide, approved by the FDA in 2003, this drug has been shown to prevent fusion and subsequent viral entry (Egelhofer et al., 2004).

## Most New Infections Are Caused by CCR5- Rather Than CXCR4-Utilizing HIV-1

Studies have shown that HIV-1 transmission involves a substantial bottleneck in the number of viral quasispecies that enter the PBMC compartment following a heterosexual genital tract transmission event with only a small number of quasispecies and often a single genotype existing in the periphery after the initial month of infection. Subsequently, genetic diversity of the HIV-1 viral quasispecies rebounds greatly during symptomatic and chronic infection prior to initiation of ART (Wolfs et al., 1992; Wolinsky et al., 1992; Zhu et al., 1993; Sagar et al., 2004). More specifically, a number of experimental assays were used to determine that up to 80% of heterosexually transmitted HIV-1 infections worldwide were established by a single HIV-1 genotype in each infected individual, known as the transmitter founder virus (Derdeyn et al., 2004; Keele et al., 2008; Abrahams et al., 2009; Haaland et al., 2009). The low diversity of transmitted virus in newly infected individuals has important implications for vaccine development, with the intention of developing an HIV-1 vaccine that could be broadly protective across the genetic diversity of transmitted/founder virus population across a large number of individuals. Interestingly, additional studies have suggested that most of the transmitter/founder viruses utilize CCR5 as the co-receptor for cell entry (Keele et al., 2008). Clinical samples from early HIV-1 infections have shown that HIV-1 variants predominantly use CCR5 exclusively during the course of HIV-1 infection (Brumme et al., 2005; Moyle et al., 2005). It is also known that specific HIV-1 strains utilize CXCR4 as the entry co-receptor (Bleul et al., 1996). However, less than 1% of infected individuals possessed HIV-1 that was exclusively CXCR4-utilizing (Brumme et al., 2005; Moyle et al., 2005). A more recent clinical trial of the CCR5 inhibitor vicriviroc demonstrated that 50% of subjects had CCR5-utilizing virus, whereas CXCR4-utilizing viruses were only found in 4% of involved subjects. The other 46% contained both CXCR4- and CCR5-utilizing viruses (Wilkin et al., 2007). These clinical studies highlight the importance of CCR5 for the course of HIV-1 infection from the point of transmission to chronic infection. It has also been implied that the stochastic process and selection pressure may lead to a shift in co-receptor usage that is distinct for each patient.

## Gene Editing to Engineer Cells Resistant to HIV-1 Infection

Research efforts aimed at curing HIV-1 infection are currently focused on two curative strategies: functional and sterilizing cures (Van Lint et al., 2013; Khalili et al., 2015, 2017). A sterilizing cure would entail the removal of provirus from all latent reservoir cells, thus curing the patient of HIV-1 altogether (Stone et al., 2013; Khalili et al., 2015, 2017). A functional cure would enable patients to suppress viral replication in the absence of ART without accomplishing complete eradication of the latent proviral reservoir (Van Lint et al., 2013; Khalili et al., 2015). Rapidly advancing techniques in gene editing offer a promising approach to both of these general curative strategies (Khalili et al., 2015, 2017). Gene editing techniques capable of highly specific excision offer a compelling approach to the development of a sterilizing cure via complete proviral removal (Manjunath et al., 2013; Dampier et al., 2014, 2017, 2018; Hu et al., 2014). Toward a functional cure, HIV-1 co-receptors CXCR4 and CCR5 are considered therapeutic targets for disruption of viral replication and modern gene editing nucleases have the capability to disrupt cell-surface co-receptor expression (Khalili et al., 2015, 2017). Enthusiasm for CCR5 as a therapeutic target has been bolstered by the long-term functional suppression of viral gene expression in the Berlin patient. The patient’s functional cure is attributed to an immune cell transplant containing the CCR5Δ32 mutation, a truncated CCR5 protein with ablated expression on the cell surface (Allers et al., 2011; Maier et al., 2013). Attempts to suppress HIV-1 co-receptors to curb viral proliferation did not begin with gene editing nucleases. RNA interference (RNAi) using short-hairpin RNAs (shRNA) was an approach used before gene editing technologies were available (Rossi et al., 2007; Shimizu et al., 2010). The lentiviral vector expressing CCR5-shRNA has been developed to transfect primary human lymphocytes using oligofectamine. The transfected cells showed 60% to 96% reduction in CCR5 expression (An et al., 2006). A study using a humanized mouse model with human hematopoietic progenitor stem cells transduced by CCR5-shRNA-expressing lentivirus showed that there was no production of p24 in an *ex vivo* test after 12 days of infection with CCR5-tropic HIV-1 NL4-3 in CCR5-shRNA+ splenocytes (Shimizu et al., 2010). However, gene editing nucleases have a distinct advantage over RNAi techniques. RNAi must be continuously expressed in order to continuously suppress CCR5 expression on the cell surface. Gene editing nucleases need only be expressed transiently in order to disrupt CCR5 expression indefinitely (Holt et al., 2010).

## Zinc Finger Nucleases: Targeting CCR5

Zinc finger nucleases (ZFNs) emerged early in the field of gene editing with the novel capability to target specific genomic sites (Bibikova et al., 2003; Porteus and Baltimore, 2003; Urnov et al., 2005; Beumer et al., 2006). Modification of ZFNs introduced a structural requirement such that the nucleases could only cleave when paired as a heterodimer which improved specificity of ZFN sequence targeting (Miller et al., 2007). The progression of this technology naturally led to attempts to edit the genes expressing CCR5 in CD4+ T cells. Early successes, demonstrating the feasibility of this approach have focused on specifically targeting disruption of the gene encoding CCR5 in human CD4+ T cells or hematopoietic stem/progenitor cells (HSPCs). The strategy relies on stable transplantation of modified cells into an organism and the preferential survival of modified cells when challenged with CCR5-utilizing HIV-1 virus. Multiple studies report measurable reduction of viral load following treatment in animal models as well as clinical trials (Perez et al., 2008; Holt et al., 2010; Tebas et al., 2014). Interestingly a number of the *in vitro* and *in vivo* results came after the start of clinical trials. A recent study successfully disrupted CCR5 using ZFN in primary human CD4+ T cells. Adenovirus Ad5/35 transduction was examined in HIV-1-infected patient-derived primary CD4+ T cells and revealed a ZFN-induced modification efficiency of 35.6% on CCR5 alleles (Perez et al., 2008). Mice engrafted with modified cells showed significantly reduced viral loads at 30 and 50 days compared to those engrafted with wild-type cells (Perez et al., 2008). In another study, ZFNs were used to disrupt the gene for CCR5 in human CD34 HSPC cells (Holt et al., 2010). ZFN expression plasmid was introduced through nucleofection in human hematopoietic stem cells targeting CCR5 and modified a mean of 17% of CCR5 alleles in CD34+ HSPCs (Holt et al., 2010). Mice transplanted with the modified HSPCs showed a selective advantage for successfully edited CD4^+^ T cells when infected with CCR5-utilizing HIV-1. At peak viremia, 6 weeks post-infection, mice transplanted with ZFN-modified cells showed a significantly reduced systemic viral load. Furthermore, mice that were administered a ZFN-modified transplant but were infected with a CXCR4-utilizing HIV-1 strain did not demonstrate an elevated resistance to infection. This supports the understanding that the mechanism of conferred resistance is CCR5-dependent (Holt et al., 2010). In addition, this study highlights the fact that modified CCR5-deficient HSPCs are potentially capable of replenishing the immune system with HIV-1 resistant cells providing a sustained therapeutic effect (Holt et al., 2010).

## Transcription Activator-Like Effector Nucleases: Targeting CCR5

Transcription activator-like effector nucleases (TALENs) are another class of gene editing technology being applied to HIV-1 co-receptor disruption (Ye et al., 2014; Jin et al., 2018; Yu A.Q. et al., 2018). TALENs are comprised of a non-specific nuclease, FokI, guided by customizable binding domains (Khalili et al., 2015). Some studies have found that TALENs exhibit an editing efficiency comparable to ZFNs when targeting CCR5 but caused less cytotoxicity with fewer off-target cleavage events (Mussolino et al., 2011, 2014; Khalili et al., 2015). One such study tested an array of TALENs designed to target the CCR5 gene by transfecting human primary CD4+T cells and GHOST-CCR5-CXCR4 (a reporter cell line for HIV-1 infection). The most favorable TALEN of those examined exhibited higher specificity and lower cytotoxicity than some ZFNs that have already moved to clinical trials (Perez et al., 2008; Tebas et al., 2014). In particular, CCR2 has a high degree of homology with CCR5 and sequence analysis of PCR amplicons expressing the target regions for the TALENs and ZFNs 48 h post-transfection, showed significant mutations in the CCR2 region of the ZFN-treated cells. In contrast, no mutations were detected in the TALEN-treated cell population (Shi et al., 2017). An additional study also showed that particularly high cleavage efficiency can be achieved using TALENs *in vitro* (Guilinger et al., 2014). Moreover, engineered TALENs were used to generate the CCR5Δ32 mutation *in vitro* using CD4^+^ U87 cells and achieved a cleavage efficiency greater than 50% without selective pressure (Yu A.Q. et al., 2018). Even so, there is mounting evidence that clustered regulatory interspaced short palindromic repeat (CRISPR)-associated 9 (Cas9) (CRISPR/cas9) gene editing system (discussed in more detail below) is an even more effective gene editing tool than TALENs or ZFNs (Khalili et al., 2017). A study was performed to compare these different gene editing platforms and found that CRISPR efficiency for generating the CCR5Δ32 mutation via biallelic disruption in induced pluripotent stem cells (iPSCs) was more than twice as effective when compared to TALENs (Ye et al., 2014).

## Clinical Trials of CCR5 Gene Editing

**FIGURE 2:**
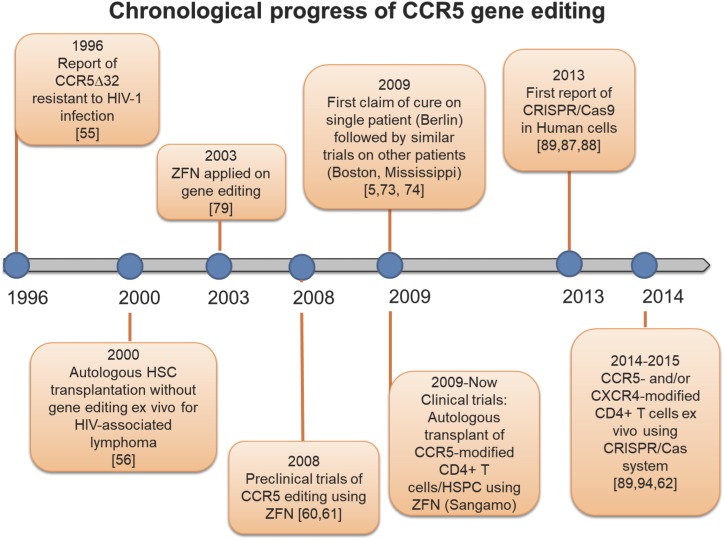
Timeline of CCR5 genetic editing. History of studies uncovering the CCR5Δ32 mutation, and landmark investigations modifying the CCR5 receptor.

Published results from clinical trials of CCR5 have primarily focused on the use of ZFN for CCR5 editing and began before a number of the mechanistic studies from ZFNs and TALENs were performed and published (Figure 2). In 1996, Samson and colleagues reported that a 32-base-pair deletion mutant of CCR5 (CCR5Δ32) confers resistance to HIV-1 infection in CD4^+^ cells (Samson et al., 1996). Early efforts to exploit the CCR5Δ32 mutation were focused on adoptively transferred CCR5Δ32 allogeneic hematopoietic stem cells as a potential therapy (Campbell et al., 1999; Gabarre et al., 2000; Krishnan et al., 2001). Previous attempts of *ex vivo* CCR5 modification on CD4^+^ T cells or HSCs have utilized a CCR5-targeted ribozyme alone or with combined RNA interference of HIV-1 viral genes (DiGiusto et al., 2010). The clinical trial demonstrated an average of 0.14% of CCR5-modified cells among the infused cell populations. Permanent DNA-level modifications of CCR5 using ZFN have been rapidly adapted in human primary T cells or HSCs (Perez et al., 2008; Holt et al., 2010). The CCR5-modified cells were engrafted in a number of mouse models and it was demonstrated that up to 50% of CCR5 alleles were genetically disrupted. The success of *in vitro* studies has led to clinical trials. Tebas et al., firstly demonstrated the ZFN-directed CCR5 gene editing on patient-derived CD4 T cells from HIV-1-infected patients (Tebas et al., 2014). They observed a 28% modification efficiency and reconstitution efficiency of 13.9% of circulating CD4^+^ T cells after transfusion. The decline in circulating CCR5-modified cells was significantly slower than endogenous T cells during the period of ART interruption, indicating that CCR5-modified cells conferred resistance to HIV-1 infection. A phase 1 study of this regimen has mostly been completed with or without additional administration of cyclophosphamide before transfusion (ClinicalTrials.gov Identifier: NCT02388594, NCT00842634, NCT01252641, NCT01044654, NCT02225665). Another clinical trial using CCR5-disrupted HSPCs followed by autologous engraftment after 2 or 3-day administration of busulfan has been estimated to be complete by July 2018 (ClinicalTrials.gov Identifier: NCT02500849). The non-randomized phase 1 study will demonstrate further potential of CCR5-modified hematopoietic CD34+ stem/progenitor cells with respect to the control of HIV-1 infection in patients. While there have been no reported side effects from knocking out CCR5, there are reports that infection with flaviviruses in a CCR5 deficient host result in a higher probability of symptomatic infection (Glass et al., 2006).

## CRISPR Targeting CCR5

Clustered regularly interspaced short palindromic repeat (CRISPR)-associated 9 (Cas9) (CRISPR/cas9) is a third class of gene editing tools. Optimization of the CRISPR/Cas9 system has progressed recently demonstrating greater specificity and cleavage efficiency than the ZFN and TALEN systems (Fu et al., 2014; Tycko et al., 2016; Khalili et al., 2017). A recent study found that CRISPR efficiency for generating the CCR5Δ32 mutation via biallelic disruption in induced pluripotent stem cells (iPSCs) was more than twice as effective when compared to TALENs (Ye et al., 2014). More recently, a study compared the performance of the TALENS and CRISPR/Cas9 systems. They concluded that CRISPR/Cas9 outperformed TALENs when targeting the same region of the CCR5 gene. CRISPR/Cas9 showed greater efficiency in both delivery and editing performance *in vitro* (Nerys-Junior et al., 2018).

The CRISPR/Cas9 system offers a distinct advantage over other gene-editing nucleases. Adaptation of CRISPR/Cas9 targeting is simple compared to adjustments to the ZFN and TALEN systems. Changing the cleavage target of CRISPR/Cas9 requires only a change in gRNA sequence. ZFN and TALEN systems require a redesigned protein binding domain in order to change cleavage target sequences (Gaj et al., 2012; Ousterout and Gersbach, 2016; Khalili et al., 2017). This becomes an important consideration from a design perspective. Overall, clinical trials involving ZFN notwithstanding, CRISPR-Cas9 has been the most promising of the gene editing technologies available for the ablation of HIV-1 co-receptors on susceptible cells for HIV-1 infection.

Additional studies have also demonstrated that CRISPR/Cas9 can be used to ablate HIV-1 co-receptor expression. CRISPR/Cas9 was delivered to CD4+ T cells *in vitro* using a lentiviral vector. Modified cells showed resistance to CCR5-utilizing virus and a selective advantage over wild-type cells (Wang et al., 2014). Several studies have also reported successful inhibition of CXCR4 expression and a resultant increase in HIV-1 resistance *in vitro* (Hou et al., 2015; Liu et al., 2017, 2018; Wang et al., 2017). Recently, a study reported the successful ablation of both the CCR5 and CXCR4 co-receptors *in vitro*. CRISPR/Cas9 mediated editing yielded biallelic disruption of both co-receptors in 9% of modified GHOST CXCR4+ CCR5+ cells (Yu S. et al., 2018).

Despite the promising aspects of gene editing in general and CRISPR/Cas9 in particular, challenges remain. CRISPR/Cas9 has shown great promise but lacks extensive animal model validation that has been achieved with ZFNs. A recent study has been reported using the CRISPR/Cas9 system to edit CCR5 in HSPCs with subsequent use of those HSPCs to engraft a humanized mouse model. CRISPR/Cas9 modified cells conferred resistance to a strain of CCR5-utilizing virus that was comparable to equivalent ZFN strategies (Xu et al., 2017). Whether the transplantation of autologous HSPC cells with nuclease-modified CCR5 and CXCR4 genes can be implemented to induce sustainable cell counts of HIV-1 resistant cells thereby precluding the need for ART for an extended period, remains to be seen. To be effective, the strategy of transplanting co-receptor deficient cells to resist propagation of HIV-1 infection must result in a sustainable population of such resistant cells. HSPCs therefore, are the most viable choice for long-term sustainable resistance because they are capable or replenishing the supply of HIV-1 resistant immune cells. However, CXCR4 has been shown to be critical for engraftment of human stem cells into mice (Peled et al., 1999).

## Zinc Finger Nucleases: Targeting CXCR4

The majority of published studies investigating HIV-1 co-receptor editing using ZFNs have focused on CCR5. However, the same approach has been applied to editing CXCR4 with some success (Yuan et al., 2012; Didigu et al., 2014). A study compared shRNA suppression of CXCR4 to targeted ZFN disruption of CXCR4 in human CD4^+^ T cells (Yuan et al., 2012). They found ZFN disruption to be the more effective technique. Although lentiviral-vector delivered shRNA was able to suppress expression of CXCR4 on the cell surface, shRNA-expressing cells remained susceptible to viral entry *in vitro*. ZFN-modified CXCR4-deficient cells, by contrast, demonstrated selective advantage when challenged with HIV-1 infection both *in vitro* and *in vivo*. These cells also conferred HIV-1 resistance to engrafted mice (Yuan et al., 2012). Another study was able to advance this technology by successfully inactivating both CCR5 and CXCR4 in human CD4^+^ T cells. The dual edited cells were shown to have a selective advantage when confronted with CCR5- and CXCR4-utilizing strains of HIV-1 both *in vitro* and after being engrafted into humanized mice (Didigu et al., 2014).

## The Possibility of Clinical Trials Involving the Use of CXCR4 Gene Editing in Humans

Although previous studies have shown great potential of CCR5 as the target of preventing HIV-1 infection, it is worthy to note that the inhibition of the CCR5-binding site or CCR5 mutants cannot prevent X4-utilizing HIV-1 from infecting cells. A recent report demonstrated a pre-existing CXCR4-utilizing HIV-1 variant in a patient that acquired an allogeneic transplantation of CCR5-knockout stem cells (Verheyen et al., 2018). The CXCR4-utilizing HIV-1 variant was shown to be highly replicative and rapidly rebounded after allogeneic transplantation. Therefore, the examination of CXCR4 modification is of importance in order to block infection by both CCR5- and CXCR4-utilizing viruses. Recently, a study generated dual-modified CCR5 and CXCR4 human primary CD4+ T cells (Didigu et al., 2014). The successful engraftment followed by the protection from CCR5- or CXCR4-utilizing HIV-1 viruses in a humanized mouse model demonstrated that the gene editing for CXCR4 may enhance the establishment of comprehensive protection from dual tropic viruses or patients carrying both R5 or X4 viruses. Another study further utilized the CRISPR/Cas9 system to increase the specificity and efficacy of CXCR4 ablation on Ghost-X4 T cells and macaque primary CD4+ T cells. The potential of CCR5-modified cell therapy can be attributed to the low toxicity of homozygous modification, which may be due to the existence of redundant chemokine receptors for each of the ligands recognized by CCR5 as previously reviewed (Mantovani, 1999). However, there might be substantial concerns to genetically modify CXCR4 with respect to hematopoietic stem cells. CXCR4 has been shown to be embryonic lethal with homozygous mutations in a murine model (Zou et al., 1998). It is also suggested that CXCR4 plays a role with respect to the retention of hematopoietic progenitor cells in the bone marrow (Broxmeyer et al., 2005). The downstream effect of CXCR4 ablation will require further examination and these studies will determine whether increased mobilization of HSC adversely affects the engraftment and reconstitution of CXCR4-modified HSCs. The major ligand for CXCR4, stromal cell-derived factor 1 (SDF-1), also known as CXCL12, can be recognized by CXCR4 (Bleul et al., 1996; Oberlin et al., 1996) and CXCR7 (Balabanian et al., 2005). An experimental study using the SDF-1 knockout mouse suggested that SDF-1 was involved in B cell development and bone-marrow myelopoiesis (Nagasawa et al., 1996). However, the downstream signaling of CXCR4 using selected heterotrimeric G proteins has been thought to be independent of CXCR7. The unique pathway facilitated by CXCL12-CXCR4 may be a major hurdle for the development of CXCR4-modified hematopoietic stem cells as the resistant cell reservoir for HIV-1 infection.

## Cell-Free Versus Cell-To-Cell Transmission

The classical CD4, CCR5, or CXCR4 axis for cell-free HIV-1 entry has been extensively studied. In most of the gene editing studies performed with CCR5 or CXCR4, the amount of viral replication quantified was primarily from cell-free virus. While being able to stop cell-free infection is important, the method of cell-to-cell transmission should not be overlooked. Described below is how the field of broadly neutralizing antibodies have had mixed success with blocking cell-to-cell transmission and how a gene editing approach may be able to overcome some of those hurdles. In addition, the section below will discuss how HIV-1 can enter target cells through bulk endocytosis or from a macrophage phagocytosing an infected dying T-cell.

One of the promising therapeutic approaches to blocking HIV-1 infection is through the use of broadly neutralizing antibodies (Huang et al., 2016; Nishimura et al., 2017; Gautam et al., 2018). The idea behind this approach is the same as a vaccine, stop the virus from entering cells. A multitude of studies have reported the successful prevention of multiple strains of virus from beginning the infection cycle (Xu et al., 2018). Now the field faces the question: can these antibodies be used to block the entirety of the quasispecies? If so, how long will the antibodies last in circulation (Badamchi-Zadeh et al., 2018)? There are reports of modifying these antibodies to persist in the plasma making them effective for a longer period of time (Gautam et al., 2018). While these reports are promising, these antibodies still degrade over time and if they fall below a certain threshold they no longer protect from HIV-1 infection. In addition, it has been shown that these broadly neutralizing antibodies have performed well neutralizing cell-free virus. However, blocking cell-to-cell transfer of virus has been much more difficult (Malbec et al., 2013; Schiffner et al., 2013). A recent study characterized the differences between a number of broadly neutralizing antibodies and their ability to block cell-to-cell transmission of HIV-1. The results described how cell-to-cell transmission was not efficiently neutralized by antibodies. This study also included transmitter founder envelopes which showed a reduced ability to be neutralized during cell-to-cell transmission (Li et al., 2017). An additional study was performed that was able to identify a few broadly neutralizing antibodies that were able to block cell-to-cell transmission to some degree (Malbec et al., 2013). These antibodies included ones that target the CD4 binding site (NIH45-46, 3BNC60), and the V3 loop (10-1074, PGT121). Antibodies that targeted the V3 loop were effective against CCR5-utilizing virus but were ineffective against CXCR4-utilizng virus. As described in the previous section, being able to use gene editing technology would allow cells to become resistant to both CCR5- and CXCR4-utilizing viruses. Moreover, the concern of antibody half-life would be avoided, and the patient wouldn’t need multiple infusions of antibody to block infection over a long period of time. While not experimentally tested, the idea of combining gene editing strategies with broad neutralizing antibodies could serve as a viable clinical option. While the broad neutralizing antibodies could neutralize cell-free virus, the gene edited cells could resist cell-to-cell transmission, blocking multiple routes of viral infection.

The ability to stop cell-to-cell transmission of HIV-1 is crucial because cell-to-cell transmission is up to 10-fold more efficient than cell-free transmission (Chen et al., 2007). Cell-to-cell transmission is in part more efficient due to the formation of a virological synapse between an infected cell and an uninfected cell (Bracq et al., 2018). This synapse forms when a donor T cells comes into contact with an infected T-cell. The cells maintain close contact through actin rearrangement and LFA-1/ICAM-1 interactions. Once gp120 from the infected cell binds to CD4 on the donor cell, a cytosolic rearrangement is triggered that sequesters CCR5 and CXCR4 to the site of cellular contact. This enables the virus to infect the donor cell in a manner very difficult to block by neutralizing antibodies. This pitfall represented another advantage for using gene editing technologies to edit the co-receptors needed for HIV-1 entry. The virological synapse will still form between two cells but without the co-receptors (CCR5 or CXCR4) gp120 will not be able to undergo the conformational changes necessary to enter the uninfected cell. While cell-to-cell transmission has been shown to be more effective than cell-free transmission *in vitro*, the *in vivo* efficacy is still debated in the field. In light of this debate there have been a number of studies that have looked at how well antiretroviral compounds are able to block cell-to-cell transmission. It was shown that a number of NNRTIs, entry inhibitors, and protease inhibitors were able to effectively block cell-to-cell transmission. Moreover, the same study demonstrated how certain NRTIs were not able to effectively block cell-to-cell transmission highlighting the need for careful consideration when deciding on the best treatment options for patients (Agosto et al., 2014). Moreover, there is considerable evidence to suggest that at the virological synapse, there are a large number of viral particles thereby increasing the probability of infection and a possible mechanism for overcoming antiretroviral therapy (Agosto et al., 2015). An additional study utilized HIV-1-infected patient sera to determine if that sera was able to neutralize cell-free and cell-to-cell transmission. It was demonstrated that while patient sera was able to effectively neutralize cell-free transmission, it was only half as effective against cell-to-cell transmission (Dale et al., 2011). While the previous studies used *in vitro* systems, there has been a study that utilized humanized mouse models to study the kinetics of HIV-1 cell-to-cell transmission. This study highlighted that in an *in vivo* system, cell-to-cell transmission was occurring frequently and was more resistant to AZT than cell-free transmission in the lung of infected mice. It was also observed that cell-free and cell-to-cell transmission yielded very similar viral loads, indicating that cell-to-cell transmission is effective *in vivo* (Law et al., 2016).

Another alternative entry mechanism for HIV-1 has been shown to be through a macrophage phagocytosing an infected T-cell (Baxter et al., 2014). It is well established that macrophages phagocytose dead or dying cells. When a T-cell has been infected by HIV-1 it normally dies within 24–48 h (Doitsh and Greene, 2016). During this window of cell death, macrophages have been shown to engulf infected T cells. HIV-1 is then able to infect the macrophage through what has been shown to be a CD4/CCR5 dependent pathway. A study showed that monocyte-derived macrophages (MDMs) that were homozygous for the CCR5Δ32 mutation were resistant to infection from engulfing infected T cells (Baxter et al., 2014). While HIV-1 has been shown to rely on CD4 and CCR5 when infecting macrophages from engulfed T cells, there is a CD4/CCR5 independent mechanism to infect macrophages. Previous studies have used the HIV-1 molecular clone NL4-3 without an envelope (NL4-3 -env) and assessed whether it could infect macrophages (Gobeil et al., 2012). Depending on the activation state of the macrophage, in this case, a classical M1 macrophage was able to become infected with an NL4-3 env while M0 and M2a macrophages showed little to no infection. It was hypothesized in this study that HIV-1 was utilizing an alternative endocytosis pathway to escape the endosome. This study also highlighted the point that while HIV-1 could gain access to activated M1 macrophages, viral replication was very limited as demonstrated by decreased luciferase expression from an NL4-3 luciferase construct. Although HIV-1 was endocytosed into the cell, escaping from the endosome proved to be very difficult.

## Conclusion and Future Directions

**FIGURE 3:**
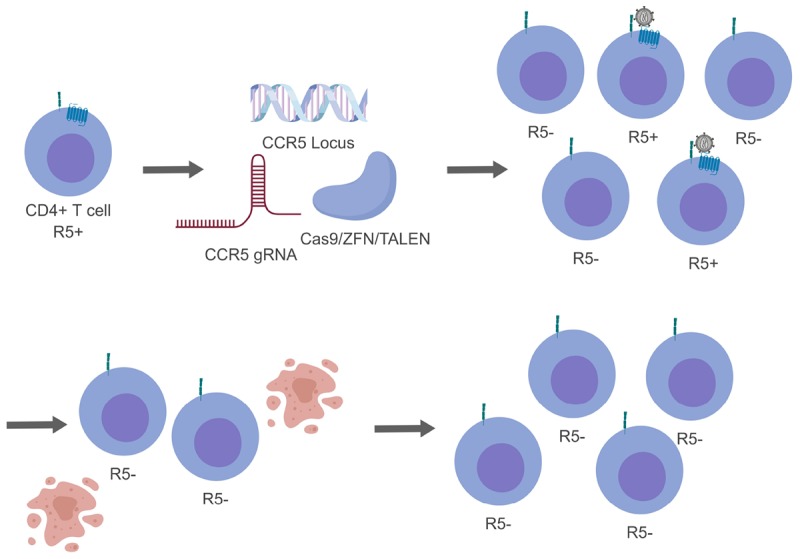
Genome editing of CCR5 resulting in a heterogeneous population. For simplicity only CCR5 gene editing has been presented. While generating modified CD4+ T cells, not all cells will be successfully edited generating a mixed population (top, right). During this time HIV-1 will be able to replicate in cells that are CCR5+ and indirectly select for cells that are CCR5- (bottom, right).

Antiretroviral therapy has transformed HIV-1 into a chronic but manageable disease. Research investigations are now focus on methods to develop a functional or sterilizing cure to prevent or further dampen HIV-1 disease. Gene editing technologies stand as a promising method to reach a functional or sterilizing cure with results both *in vitro* and *in vivo*. In addition, there are multiple clinical trials underway to evaluate how efficacious this therapy can be in human patients. While these newer approaches show promise, they are not without their own obstacles. In order to be a truly effective therapy, gene editing efficiency would need to be improved. Moreover, getting a large enough pool of successfully edited cells will be important for therapies not involving stem cells. The potential benefits of successful gene editing technologies have made them an attractive option for a next generation treatment for HIV-1 (Figure 3).

Some of the necessary challenges to transforming gene editing techniques into an attractive curative HIV-1 treatment are common to all gene editing strategies. Efficient delivery of therapy to targeted tissues and suppression of off-target effects are two such challenges. Efforts to address these hurdles are underway.

The sequence length of the ZFN module (∼1 kb) is relatively small compared with TALEN (∼3 kb) and CRISPR/Cas9 systems (∼4 kb). This has been shown to be advantageous for vector-based delivery. However, limited DNA targets due to the restricted combination of Zinc finger protein (ZFP) and cytotoxicity due to off-target cleavages has hindered the development of ZFN-mediated therapy. Although TALEN has reduced cytotoxicity and increased design flexibility compared to ZFN, the size of TALEN has been a major challenge for vector-based delivery. Nonetheless, the rearrangement of the sequence recognition site on ZFN and TALEN for different target loci has been considered time-consuming and complicated. This complex process has provided a clear advantage to RNA-guided gene editing systems with respect to anti-HIV-1 therapeutic development. By changing the protospacer region on a sgRNA, the CRISPR/Cas9 system can be versatile enough to target most of the desired sequences in HIV-1 genomes. However, the size of SpCas9 (∼4 kb) has been a predominant issue for vector-based delivery. The suitable size of SaCas9 (∼3 kb) facilitates more efficient delivery into viral vectors that have a limited storage capacity [Wang et al., 2017]. This study has successfully edited CXCR4 in CD4^+^ T cells using *Staphylococcus aureus* Cas9 (SaCas9) and a lentiviral delivery system. Modified cells showed resistance to HIV-1 infection, which has provided a promising stepping stone for *in vivo* modification.

An additional challenge for any kind of gene editing strategy to target HIV-1 is centered on being able to locate and modify cells in different anatomical sites or modify precursor cells that are destined to traffic to tissue sites. This is an especially important consideration because the latent reservoir that HIV-1 develops has been shown to be located in multiple anatomical sites throughout the body. A recent study performed in rhesus macaques (RM) with SIV has illustrated that even when ART was administered there were sites of viral RNA-positive cellular staining in multiple organ systems (Estes et al., 2017; Deleage et al., 2018). For any *in vivo* gene editing approach, a delivery system that can be transported to multiple sites will be highly beneficial. In addition, editing tissue resident cells will be vital to stopping reactivated virus from replicating and expanding the reactivation event. While gene editing systems have been used *in vivo*, it hasn’t been fully elucidated whether tissue resident cells have been successfully edited, although it has been shown that editing hematopoietic stem/progenitor cells (HSPCs) with a ZFN targeting CCR5 were able to engraft into multiple tissue compartments. These edited cells were then able to re-fill virus depleted CD4+ central memory T cells in the gut of infected NHPs (Peterson et al., 2018).

As it is currently developing, co-receptor editing for HIV-1 infection will likely not target all latent viral reservoirs, nor the entirety of the peripheral blood reservoir. Tissue resident cells for example would be less likely to be effectively targeted. Furthermore, it will probably not be able to efficiently target all potential mechanisms of viral propagation within the peripheral blood reservoir. As such, concerns about the efficacy of this treatment as a curative strategy are valid. However, one could envision that co-receptor editing could be destined to be developed into a complimentary therapeutic approach, further restricting the replication of HIV-1 in conjunction with existing ART regimens and other forms of anti-HIV-1 CRISPR/Cas9 therapeutic strategies. However, pursuit of this technology as a curative strategy remains optimistic. The cautiously accepted cure of the “Berlin Patient” has been regarded as a direct result of a CCR5-deficient bone marrow transplant. In the absence of a deeper understanding of the mechanism for the “cure” of the “Berlin Patient,” ablation of the CCR5 receptor in CD4 T cells has become the focal point of research in this area of investigation. Why the absence of the CCR5 receptor in transplanted tissue should result in non-progressive HIV-1 replication for this patient remains an interesting question. Reproducing this medical outcome remains a critical step toward finding an answer.

One approach to the reduction of off-target effects has been to directly deliver Cas9/sgRNA ribonucleoproteins (Cas9 RNPs) to infected cells rather than delivering plasmids. Delivery of the Cas9 RNPs addresses the potential for unwanted CRISPR/Cas9 activity after its intended substrate is consumed. The studies focused on Cas9 protein engineering have provided substantial improvements to minimize off-target effects. A series of SpCas9 mutants have been identified that facilitate reduced off-target effects on undesired loci including a D1135E variant, eSpCas9 and SpCas9-HF1 (Kleinstiver et al., 2015, 2016; Slaymaker et al., 2016). However, a fitness cost of on-target efficiency has been found on most of these engineered proteins. A recent report that examined the RNP delivery method with R691A SpCas9 mutant in human HSPCs showed minimal off-target editing while retaining optimal on-target activity (Vakulskas et al., 2018). Cas9 RNPs provide a distinct advantage because once they have bound and cleaved their target they are degraded, allowing for maximization of on target cleavage while minimizing off target effects. Cas9 RNPs were used to disrupt CXCR4 expression in human CD4+ T cells *in vitro* (Schumann et al., 2015). There have also been reports of using nanoparticles for delivery of Cas9-RNPs into cells (Mout et al., 2017).

Minimization of the number of off-target cleavage events has been of paramount importance in gene editing. In this regard, we are not aware of any high-throughput studies that have demonstrated any off-target cleavage events during CCR5 ablation. Furthermore, to date, we are not aware of any studies that have indicated any downstream off-target effects of off-target cleavage events (e.g., cell death) at a population level for treated cells. Even so, careful screening for off-target cleavage will continue to be important in this context. Even in an *ex vivo* treatment, the changes introduced in cells will be sustained in modified cells during subsequent cultivation *in vitro* or after transplanted cells into experimental animals. Furthermore, any broadly applicable treatment dependent on sequence similarity will demand consideration of genetic diversity for large scale application. Techniques such as recently published unbiased sequencing techniques capable of detecting the sequence location of cleavage events in targeted cells will be critical to validate gene editing therapies (Tsai et al., 2015, 2017).

There have been important recent advances in therapeutic strategies focused on ablation of the HIV-1 receptor and co-receptors but there is still much to be done to refine this approach with respect to a reliable therapeutic application. It is likely that targeting the receptors to prevent acute viral infection or minimize HIV-1 disease emanating from activation of latent virus within tissue reservoirs will require a combined approach with other strategies that engage a more robust immune response in conjunction with targeting existing or yet to be identified aspects of the viral life cycle.

## Author Contributions

AGA, MN, and BW conceptualized and outlined the manuscript. AGA, C-HC, and AA wrote the first draft of the manuscript. All authors edited and approved the final version.

## Conflict of Interest Statement

The authors declare that the research was conducted in the absence of any commercial or financial relationships that could be construed as a potential conflict of interest.

## References

[B1] Abrahams M. R. Anderson J. A. Giorgi E. E. Seoighe C. Mlisana K. Ping L. H. (2009). Quantitating the multiplicity of infection with human immunodeficiency virus type 1 subtype C reveals a non-poisson distribution of transmitted variants. *J. Virol.* 83 3556–3567. 10.1128/JVI.02132-08 19193811 PMC2663249

[B2] Agosto L. M. Uchil P. D. Mothes W. (2015). HIV cell-to-cell transmission: effects on pathogenesis and antiretroviral therapy. *Trends Microbiol.* 23 289–295. 10.1016/j.tim.2015.02.003 25766144 PMC4417442

[B3] Agosto L. M. Zhong P. Munro J. Mothes W. (2014). Highly active antiretroviral therapies are effective against HIV-1 cell-to-cell transmission. *PLoS Pathog.* 10:e1003982. 10.1371/journal.ppat.1003982 24586176 PMC3937346

[B4] Alkhatib G. Combadiere C. Broder C. C. Feng Y. Kennedy P. E. Murphy P. M. (1996). CC CKR5: a RANTES, MIP-1alpha, MIP-1beta receptor as a fusion cofactor for macrophage-tropic HIV-1. *Science* 272 1955–1958. 10.1126/science.272.5270.1955 8658171

[B5] Allers K. Hutter G. Hofmann J. Loddenkemper C. Rieger K. Thiel E. (2011). Evidence for the cure of HIV infection by CCR5-Delta32/Delta32 stem cell transplantation. *Blood* 117 2791–2799. 10.1182/blood-2010-09-309591 21148083

[B6] An D. S. Qin F. X. Auyeung V. C. Mao S. H. Kung S. K. Baltimore D. (2006). Optimization and functional effects of stable short hairpin RNA expression in primary human lymphocytes via lentiviral vectors. *Mol. Ther.* 14 494–504. 10.1016/j.ymthe.2006.05.015 16844419 PMC2562632

[B7] Arainga M. Edagwa B. Mosley R. L. Poluektova L. Y. Gorantla S. Gendelman H. E. (2017). A mature macrophage is a principal HIV-1 cellular reservoir in humanized mice after treatment with long acting antiretroviral therapy. *Retrovirology* 14:17. 10.1186/s12977-017-0344-7 28279181 PMC5345240

[B8] Badamchi-Zadeh A. Tartaglia L. J. Abbink P. Bricault C. A. Liu P. T. Boyd M. (2018). Therapeutic efficacy of vectored PGT121 gene delivery in HIV-1-infected humanized mice. *J. Virol.* 92:e01925-17. 10.1128/JVI.01925-17 29321310 PMC5972893

[B9] Balabanian K. Lagane B. Infantino S. Chow K. Y. Harriague J. Moepps B. (2005). The chemokine SDF-1/CXCL12 binds to and signals through the orphan receptor RDC1 in T lymphocytes. *J. Biol. Chem.* 280 35760–35766. 10.1074/jbc.M508234200 16107333

[B10] Baxter A. E. Russell R. A. Duncan C. J. Moore M. D. Willberg C. B. Pablos J. L. (2014). Macrophage infection via selective capture of HIV-1-infected CD4^+^ T cells. *Cell Host Microbe* 16 711–721. 10.1016/j.chom.2014.10.010 25467409 PMC4271767

[B11] Beumer K. Bhattacharyya G. Bibikova M. Trautman J. K. Carroll D. (2006). Efficient gene targeting in Drosophila with zinc-finger nucleases. *Genetics* 172 2391–2403. 10.1534/genetics.105.052829 16452139 PMC1456366

[B12] Bibikova M. Beumer K. Trautman J. K. Carroll D. (2003). Enhancing gene targeting with designed zinc finger nucleases. *Science* 300:764. 10.1126/science.1079512 12730594

[B13] Bleul C. C. Farzan M. Choe H. Parolin C. Clark-Lewis I. Sodroski J. (1996). The lymphocyte chemoattractant SDF-1 is a ligand for LESTR/fusin and blocks HIV-1 entry. *Nature* 382 829–833. 10.1038/382829a0 8752280

[B14] Bracq L. Xie M. Benichou S. Bouchet J. (2018). Mechanisms for cell-to-cell transmission of HIV-1. *Front. Immunol.* 9:260. 10.3389/fimmu.2018.00260 29515578 PMC5825902

[B15] Broxmeyer H. E. Orschell C. M. Clapp D. W. Hangoc G. Cooper S. Plett P. A. (2005). Rapid mobilization of murine and human hematopoietic stem and progenitor cells with AMD3100, a CXCR4 antagonist. *J. Exp. Med.* 201 1307–1318. 10.1084/jem.20041385 15837815 PMC2213145

[B16] Brumme Z. L. Goodrich J. Mayer H. B. Brumme C. J. Henrick B. M. Wynhoven B. (2005). Molecular and clinical epidemiology of CXCR4-using HIV-1 in a large population of antiretroviral-naive individuals. *J. Infect. Dis.* 192 466–474. 10.1086/431519 15995960

[B17] Campbell P. Iland H. Gibson J. Joshua D. (1999). Syngeneic stem cell transplantation for HIV-related lymphoma. *Br. J. Haematol.* 105 795–798. 10.1046/j.1365-2141.1999.01422.x 10354149

[B18] Catalone B. J. Kish-Catalone T. M. Budgeon L. R. Neely E. B. Ferguson M. Krebs F. C. (2004). Mouse model of cervicovaginal toxicity and inflammation for preclinical evaluation of topical vaginal microbicides. *Antimicrob. Agents Chemother.* 48 1837–1847. 10.1128/AAC.48.5.1837-1847.2004 15105142 PMC400576

[B19] Chan D. C. Fass D. Berger J. M. Kim P. S. (1997). Core structure of gp41 from the HIV envelope glycoprotein. *Cell* 89 263–273. 10.1016/S0092-8674(00)80205-6 9108481

[B20] Chen P. Hubner W. Spinelli M. A. Chen B. K. (2007). Predominant mode of human immunodeficiency virus transfer between T cells is mediated by sustained Env-dependent neutralization-resistant virological synapses. *J. Virol.* 81 12582–12595. 10.1128/JVI.00381-07 17728240 PMC2169007

[B21] Choe H. Farzan M. Sun Y. Sullivan N. Rollins B. Ponath P. D. (1996). The beta-chemokine receptors CCR3 and CCR5 facilitate infection by primary HIV-1 isolates. *Cell* 85 1135–1148. 10.1016/S0092-8674(00)81313-6 8674119

[B22] Dale B. M. McNerney G. P. Thompson D. L. Hubner W. de Los Reyes K. Chuang F. Y. (2011). Cell-to-cell transfer of HIV-1 via virological synapses leads to endosomal virion maturation that activates viral membrane fusion. *Cell Host Microbe* 10 551–562. 10.1016/j.chom.2011.10.015 22177560 PMC3278276

[B23] Dampier W. Nonnemacher M. R. Sullivan N. T. Jacobson J. M. Wigdahl B. (2014). HIV excision utilizing CRISPR/Cas9 technology: attacking the proviral quasispecies in reservoirs to achieve a cure. *MOJ Immunol.* 1:00022. 25893217 10.15406/moji.2014.01.00022 PMC4399856

[B24] Dampier W. Sullivan N. T. Chung C. H. Mell J. C. Nonnemacher M. R. Wigdahl B. (2017). Designing broad-spectrum anti-HIV-1 gRNAs to target patient-derived variants. *Sci. Rep.* 7:14413. 10.1038/s41598-017-12612-z 29089503 PMC5663707

[B25] Dampier W. Sullivan N. T. Mell J. C. Pirrone V. Ehrlich G. Allen A. G. (2018). Broad spectrum and personalized gRNAs for CRISPR/Cas9 HIV-1 therapeutics. *AIDS Res. Hum. Retroviruses* 34 950–960. 10.1089/AID.2017.0274 29968495 PMC6238604

[B26] Deeks S. G. Wagner B. Anton P. A. Mitsuyasu R. T. Scadden D. T. Huang C. (2002). A phase II randomized study of HIV-specific T-cell gene therapy in subjects with undetectable plasma viremia on combination antiretroviral therapy. *Mol. Ther.* 5 788–797. 10.1006/mthe.2002.0611 12027564

[B27] Deleage C. Chan C. N. Busman-Sahay K. Estes J. D. (2018). Next-generation in situ hybridization approaches to define and quantify HIV and SIV reservoirs in tissue microenvironments. *Retrovirology* 15:4. 10.1186/s12977-017-0387-9 29316956 PMC5761108

[B28] Deng H. Liu R. Ellmeier W. Choe S. Unutmaz D. Burkhart M. (1996). Identification of a major co-receptor for primary isolates of HIV-1. *Nature* 381 661–666. 10.1038/381661a0 8649511

[B29] Derdeyn C. A. Decker J. M. Bibollet-Ruche F. Mokili J. L. Muldoon M. Denham S. A. (2004). Envelope-constrained neutralization-sensitive HIV-1 after heterosexual transmission. *Science* 303 2019–2022. 10.1126/science.1093137 15044802

[B30] Didigu C. A. Wilen C. B. Wang J. Duong J. Secreto A. J. Danet G. A. (2014). Simultaneous zinc-finger nuclease editing of the HIV coreceptors ccr5 and cxcr4 protects CD4^+^ T cells from HIV-1 infection. *Blood* 123 61–69. 10.1182/blood-2013-08-521229 24162716 PMC3879906

[B31] DiGiusto D. L. Krishnan A. Li L. Li H. Li S. Rao A. (2010). RNA-based gene therapy for HIV with lentiviral vector-modified CD34^+^ cells in patients undergoing transplantation for AIDS-related lymphoma. *Sci. Transl. Med.* 2:36ra43. 10.1126/scitranslmed.3000931 20555022 PMC3130552

[B32] Doitsh G. Greene W. C. (2016). Dissecting how CD4 T cells are lost during HIV infection. *Cell Host Microbe* 19 280–291. 10.1016/j.chom.2016.02.012 26962940 PMC4835240

[B33] Doranz B. J. Rucker J. Yi Y. Smyth R. J. Samson M. Peiper S. C. (1996). A dual-tropic primary HIV-1 isolate that uses fusin and the beta-chemokine receptors CKR-5, CKR-3, and CKR-2b as fusion cofactors. *Cell* 85 1149–1158. 10.1016/S0092-8674(00)81314-8 8674120

[B34] Doudna J. A. Charpentier E. (2014). Genome editing. The new frontier of genome engineering with CRISPR-Cas9. *Science* 346:1258096. 10.1126/science.1258096 25430774

[B35] Dragic T. Litwin V. Allaway G. P. Martin S. R. Huang Y. Nagashima K. A. (1996). HIV-1 entry into CD4^+^ cells is mediated by the chemokine receptor CC-CKR-5. *Nature* 381 667–673. 10.1038/381667a0 8649512

[B36] Egelhofer M. Brandenburg G. Martinius H. Schult-Dietrich P. Melikyan G. Kunert R. (2004). Inhibition of human immunodeficiency virus type 1 entry in cells expressing gp41-derived peptides. *J. Virol.* 78 568–575. 10.1128/JVI.78.2.568-575.2004 14694088 PMC368739

[B37] Estes J. D. Kityo C. Ssali F. Swainson L. Makamdop K. N. Del G. Q. (2017). Defining total-body AIDS-virus burden with implications for curative strategies. *Nat. Med.* 23 1271–1276. 10.1038/nm.4411 28967921 PMC5831193

[B38] Feng Y. Broder C. C. Kennedy P. E. Berger E. A. (1996). HIV-1 entry cofactor: functional cDNA cloning of a seven-transmembrane, G protein-coupled receptor. *Science* 272 872–877. 10.1126/science.272.5263.872 8629022

[B39] Fu Y. Sander J. D. Reyon D. Cascio V. M. Joung J. K. (2014). Improving CRISPR-Cas nuclease specificity using truncated guide RNAs. *Nat. Biotechnol.* 32 279–284. 10.1038/nbt.2808 24463574 PMC3988262

[B40] Furuta R. A. Wild C. T. Weng Y. Weiss C. D. (1998). Capture of an early fusion-active conformation of HIV-1 gp41. *Nat. Struct. Biol.* 5 276–279. 10.1038/nsb0498-276 9546217

[B41] Gabarre J. Azar N. Autran B. Katlama C. Leblond V. (2000). High-dose therapy and autologous haematopoietic stem-cell transplantation for HIV-1-associated lymphoma. *Lancet* 355 1071–1072. 10.1016/S0140-6736(00)02041-9 10744095

[B42] Gaj T. Guo J. Kato Y. Sirk S. J. Barbas CF 3rd (2012). Targeted gene knockout by direct delivery of zinc-finger nuclease proteins. *Nat. Methods* 9 805–807. 10.1038/nmeth.2030 22751204 PMC3424280

[B43] Gautam R. Nishimura Y. Gaughan N. Gazumyan A. Schoofs T. Buckler-White A. (2018). A single injection of crystallizable fragment domain-modified antibodies elicits durable protection from SHIV infection. *Nat. Med.* 24 610–616. 10.1038/s41591-018-0001-2 29662199 PMC5989326

[B44] Glass W. G. McDermott D. H. Lim J. K. Lekhong S. Yu S. F. Frank W. A. (2006). CCR5 deficiency increases risk of symptomatic West Nile virus infection. *J. Exp. Med.* 203 35–40. 10.1084/jem.20051970 16418398 PMC2118086

[B45] Gobeil L. A. Lodge R. Tremblay M. J. (2012). Differential HIV-1 endocytosis and susceptibility to virus infection in human macrophages correlate with cell activation status. *J. Virol.* 86 10399–10407. 10.1128/JVI.01051-12 22787228 PMC3457310

[B46] Guilinger J. P. Pattanayak V. Reyon D. Tsai S. Q. Sander J. D. Joung J. K. (2014). Broad specificity profiling of TALENs results in engineered nucleases with improved DNA-cleavage specificity. *Nat. Methods* 11 429–435. 10.1038/nmeth.2845 24531420 PMC4010127

[B47] Gummuluru S. Pina Akiyama N. G. Ramirez H. (2014). CD169-dependent cell-associated HIV-1 transmission: a driver of virus dissemination. *J. Infect. Dis.* 210(Suppl. 3), S641–S647. 10.1093/infdis/jiu442 25414418 PMC4303078

[B48] Haaland R. E. Hawkins P. A. Salazar-Gonzalez J. Johnson A. Tichacek A. Karita E. (2009). Inflammatory genital infections mitigate a severe genetic bottleneck in heterosexual transmission of subtype A and C HIV-1. *PLoS Pathog.* 5:e1000274. 10.1371/journal.ppat.1000274 19165325 PMC2621345

[B49] Harouse J. M. Kunsch C. Hartle H. T. Laughlin M. A. Hoxie J. A. Wigdahl B. (1989). CD4-independent infection of human neural cells by human immunodeficiency virus type 1. *J. Virol.* 63 2527–2533. 2786088 10.1128/jvi.63.6.2527-2533.1989 PMC250718

[B50] Holt N. Wang J. Kim K. Friedman G. Wang X. Taupin V. (2010). Human hematopoietic stem/progenitor cells modified by zinc-finger nucleases targeted to CCR5 control HIV-1 in vivo. *Nat. Biotechnol.* 28 839–847. 10.1038/nbt.1663 20601939 PMC3080757

[B51] Hou P. Chen S. Wang S. Yu X. Chen Y. Jiang M. (2015). Genome editing of CXCR4 by CRISPR/cas9 confers cells resistant to HIV-1 infection. *Sci. Rep.* 5:15577. 10.1038/srep15577 26481100 PMC4612538

[B52] Hu W. Kaminski R. Yang F. Zhang Y. Cosentino L. Li F. (2014). RNA-directed gene editing specifically eradicates latent and prevents new HIV-1 infection. *Proc. Natl. Acad. Sci. U.S.A.* 111 11461–11466. 10.1073/pnas.1405186111 25049410 PMC4128125

[B53] Huang J. Kang B. H. Ishida E. Zhou T. Griesman T. Sheng Z. (2016). Identification of a CD4-binding-site antibody to HIV that evolved near-pan neutralization breadth. *Immunity* 45 1108–1121. 10.1016/j.immuni.2016.10.027 27851912 PMC5770152

[B54] Huang S. H. Ren Y. Thomas A. S. Chan D. Mueller S. Ward A. R. (2018). Latent HIV reservoirs exhibit inherent resistance to elimination by CD8^+^ T cells. *J. Clin. Invest.* 128 876–889. 10.1172/JCI97555 29355843 PMC5785246

[B55] Jabalameli H. R. Zahednasab H. Karimi-Moghaddam A. Jabalameli M. R. (2015). Zinc finger nuclease technology: advances and obstacles in modelling and treating genetic disorders. *Gene* 558 1–5. 10.1016/j.gene.2014.12.044 25536166

[B56] Jin L. Deng Y. He N. Wang L. Weng M. (2018). Polyethylenimine-mediated CCR5 gene knockout using transcription activator-like effector nucleases. *J. Biomed. Nanotechnol.* 14 546–552. 10.1166/jbn.2018.2545 29663926

[B57] Joung J. K. Sander J. D. (2013). TALENs: a widely applicable technology for targeted genome editing. *Nat. Rev. Mol. Cell Biol.* 14 49–55. 10.1038/nrm3486 23169466 PMC3547402

[B58] Keele B. F. Giorgi E. E. Salazar-Gonzalez J. F. Decker J. M. Pham K. T. Salazar M. G. (2008). Identification and characterization of transmitted and early founder virus envelopes in primary HIV-1 infection. *Proc. Natl. Acad. Sci. U.S.A.* 105 7552–7557. 10.1073/pnas.0802203105 18490657 PMC2387184

[B59] Khalili K. Kaminski R. Gordon J. Cosentino L. Hu W. (2015). Genome editing strategies: potential tools for eradicating HIV-1/AIDS. *J. Neurovirol.* 21 310–321. 10.1007/s13365-014-0308-9 25716921 PMC4433555

[B60] Khalili K. White M. K. Jacobson J. M. (2017). Novel AIDS therapies based on gene editing. *Cell. Mol. Life Sci.* 74 2439–2450. 10.1007/s00018-017-2479-z 28210784 PMC5474186

[B61] Kleinstiver B. P. Pattanayak V. Prew M. S. Tsai S. Q. Nguyen N. T. Zheng Z. (2016). High-fidelity CRISPR-Cas9 nucleases with no detectable genome-wide off-target effects. *Nature* 529 490–495. 10.1038/nature16526 26735016 PMC4851738

[B62] Kleinstiver B. P. Prew M. S. Tsai S. Q. Topkar V. V. Nguyen N. T. Zheng Z. (2015). Engineered CRISPR-Cas9 nucleases with altered PAM specificities. *Nature* 523 481–485. 10.1038/nature14592 26098369 PMC4540238

[B63] Krishnan A. Molina A. Zaia J. Nademanee A. Kogut N. Rosenthal J. (2001). Autologous stem cell transplantation for HIV-associated lymphoma. *Blood* 98 3857–3859. 10.1182/blood.V98.13.3857 11739198

[B64] Kunsch C. Hartle H. T. Wigdahl B. (1989). Infection of human fetal dorsal root ganglion glial cells with human immunodeficiency virus type 1 involves an entry mechanism independent of the CD4 T4A epitope. *J. Virol.* 63 5054–5061. 2479771 10.1128/jvi.63.12.5054-5061.1989 PMC251166

[B65] Kwong P. D. Wyatt R. Robinson J. Sweet R. W. Sodroski J. Hendrickson W. A. (1998). Structure of an HIV gp120 envelope glycoprotein in complex with the CD4 receptor and a neutralizing human antibody. *Nature* 393 648–659. 10.1038/31405 9641677 PMC5629912

[B66] Law K. M. Komarova N. L. Yewdall A. W. Lee R. K. Herrera O. L. Wodarz D. (2016). In vivo HIV-1 cell-to-cell transmission promotes multicopy micro-compartmentalized infection. *Cell Rep.* 15 2771–2783. 10.1016/j.celrep.2016.05.059 27292632

[B67] Li H. Zony C. Chen P. Chen B. K. (2017). Reduced potency and incomplete neutralization of broadly neutralizing antibodies against cell-to-cell transmission of HIV-1 with transmitted founder Envs. *J. Virol.* 91 e02425-16. 10.1128/JVI.02425-16 28148796 PMC5391450

[B68] Liu S. Wang Q. Yu X. Li Y. Guo Y. Liu Z. (2018). HIV-1 inhibition in cells with CXCR4 mutant genome created by CRISPR-Cas9 and piggyBac recombinant technologies. *Sci. Rep.* 8:8573. 10.1038/s41598-018-26894-4 29872154 PMC5988798

[B69] Liu Z. Chen S. Jin X. Wang Q. Yang K. Li C. (2017). Genome editing of the HIV co-receptors CCR5 and CXCR4 by CRISPR-Cas9 protects CD4^+^ T cells from HIV-1 infection. *Cell Biosci.* 7:47. 10.1186/s13578-017-0174-2 28904745 PMC5591563

[B70] Maartens G. Celum C. Lewin S. R. (2014). HIV infection: epidemiology, pathogenesis, treatment, and prevention. *Lancet* 384 258–271. 10.1016/S0140-6736(14)60164-1 24907868

[B71] Maddon P. J. Dalgleish A. G. McDougal J. S. Clapham P. R. Weiss R. A. Axel R. (1986). The T4 gene encodes the AIDS virus receptor and is expressed in the immune system and the brain. *Cell* 47 333–348. 10.1016/0092-8674(86)90590-8 3094962

[B72] Maier D. A. Brennan A. L. Jiang S. Binder-Scholl G. K. Lee G. Plesa G. (2013). Efficient clinical scale gene modification via zinc finger nuclease-targeted disruption of the HIV co-receptor CCR5. *Hum. Gene Ther.* 24 245–258. 10.1089/hum.2012.172 23360514 PMC3609630

[B73] Malbec M. Porrot F. Rua R. Horwitz J. Klein F. Halper-Stromberg A. (2013). Broadly neutralizing antibodies that inhibit HIV-1 cell to cell transmission. *J. Exp. Med.* 210 2813–2821. 10.1084/jem.20131244 24277152 PMC3865481

[B74] Manjunath N. Yi G. Dang Y. Shankar P. (2013). Newer gene editing technologies toward HIV gene therapy. *Viruses* 5 2748–2766. 10.3390/v5112748 24284874 PMC3856413

[B75] Mantovani A. (1999). The chemokine system: redundancy for robust outputs. *Immunol. Today* 20 254–257. 10.1016/S0167-5699(99)01469-3 10354549

[B76] Markosyan R. M. Cohen F. S. Melikyan G. B. (2003). HIV-1 envelope proteins complete their folding into six-helix bundles immediately after fusion pore formation. *Mol. Biol. Cell* 14 926–938. 10.1091/mbc.e02-09-0573 12631714 PMC151570

[B77] McDonald D. Wu L. Bohks S. M. KewalRamani V. N. Unutmaz D. Hope T. J. (2003). Recruitment of HIV and its receptors to dendritic cell-T cell junctions. *Science* 300 1295–1297. 10.1126/science.1084238 12730499

[B78] Miller J. C. Holmes M. C. Wang J. Guschin D. Y. Lee Y. L. Rupniewski I. (2007). An improved zinc-finger nuclease architecture for highly specific genome editing. *Nat. Biotechnol.* 25 778–785. 10.1038/nbt1319 17603475

[B79] Mitsuyasu R. T. Anton P. A. Deeks S. G. Scadden D. T. Connick E. Downs M. T. (2000). Prolonged survival and tissue trafficking following adoptive transfer of CD4zeta gene-modified autologous CD4^+^ and CD8^+^ T cells in human immunodeficiency virus-infected subjects. *Blood* 96 785–793. 10910888

[B80] Mout R. Ray M. Yesilbag Tonga G. Lee Y. W. Tay T. Sasaki K. (2017). Direct cytosolic delivery of CRISPR/Cas9-ribonucleoprotein for efficient gene editing. *ACS Nano* 11 2452–2458. 10.1021/acsnano.6b07600 28129503 PMC5848212

[B81] Moyle G. J. Wildfire A. Mandalia S. Mayer H. Goodrich J. Whitcomb J. (2005). Epidemiology and predictive factors for chemokine receptor use in HIV-1 infection. *J. Infect. Dis.* 191 866–872. 10.1086/428096 15717260

[B82] Mussolino C. Alzubi J. Fine E. J. Morbitzer R. Cradick T. J. Lahaye T. (2014). TALENs facilitate targeted genome editing in human cells with high specificity and low cytotoxicity. *Nucleic Acids Res.* 42 6762–6773. 10.1093/nar/gku305 24792154 PMC4041469

[B83] Mussolino C. Morbitzer R. Lutge F. Dannemann N. Lahaye T. Cathomen T. (2011). A novel TALE nuclease scaffold enables high genome editing activity in combination with low toxicity. *Nucleic Acids Res.* 39 9283–9293. 10.1093/nar/gkr597 21813459 PMC3241638

[B84] Nagasawa T. Hirota S. Tachibana K. Takakura N. Nishikawa S. Kitamura Y. (1996). Defects of B-cell lymphopoiesis and bone-marrow myelopoiesis in mice lacking the CXC chemokine PBSF/SDF-1. *Nature* 382 635–638. 10.1038/382635a0 8757135

[B85] Nerys-Junior A. Braga-Dias L. P. Pezzuto P. Cotta-de-Almeida V. Tanuri A. (2018). Comparison of the editing patterns and editing efficiencies of TALEN and CRISPR-Cas9 when targeting the human CCR5 gene. *Genet. Mol. Biol.* 41 167–179. 10.1590/1678-4685-GMB-2017-0065 29583154 PMC5901495

[B86] Nishimura Y. Gautam R. Chun T. W. Sadjadpour R. Foulds K. E. Shingai M. (2017). Early antibody therapy can induce long-lasting immunity to SHIV. *Nature* 543 559–563. 10.1038/nature21435 28289286 PMC5458531

[B87] Oberlin E. Amara A. Bachelerie F. Bessia C. Virelizier J. L. Arenzana-Seisdedos F. (1996). The CXC chemokine SDF-1 is the ligand for LESTR/fusin and prevents infection by T-cell-line-adapted HIV-1. *Nature* 382 833–835. 10.1038/382833a0 8752281

[B88] Ousterout D. G. Gersbach C. A. (2016). The development of TALE nucleases for biotechnology. *Methods Mol. Biol.* 1338 27–42. 10.1007/978-1-4939-2932-0_3 26443211 PMC5316914

[B89] Passic S. R. Ferguson M. L. Catalone B. J. Kish-Catalone T. Kholodovych V. Zhu W. (2010). Structure-activity relationships of polybiguanides with activity against human immunodeficiency virus type 1. *Biomed. Pharmacother.* 64 723–732. 10.1016/j.biopha.2010.10.001 21106331 PMC3776307

[B90] Peled A. Petit I. Kollet O. Magid M. Ponomaryov T. Byk T. (1999). Dependence of human stem cell engraftment and repopulation of NOD/SCID mice on CXCR4. *Science* 283 845–848. 10.1126/science.283.5403.845 9933168

[B91] Perez E. E. Wang J. Miller J. C. Jouvenot Y. Kim K. A. Liu O. (2008). Establishment of HIV-1 resistance in CD4^+^ T cells by genome editing using zinc-finger nucleases. *Nat. Biotechnol.* 26 808–816. 10.1038/nbt1410 18587387 PMC3422503

[B92] Peterson C. W. Wang J. Deleage C. Reddy S. Kaur J. Polacino P. (2018). Differential impact of transplantation on peripheral and tissue-associated viral reservoirs: implications for HIV gene therapy. *PLoS Pathog.* 14:e1006956. 10.1371/journal.ppat.1006956 29672640 PMC5908070

[B93] Porteus M. H. Baltimore D. (2003). Chimeric nucleases stimulate gene targeting in human cells. *Science* 300:763. 10.1126/science.1078395 12730593

[B94] Rerks-Ngarm S. Pitisuttithum P. Nitayaphan S. Kaewkungwal J. Chiu J. Paris R. (2009). Vaccination with ALVAC and AIDSVAX to prevent HIV-1 infection in Thailand. *N. Engl. J. Med.* 361 2209–2220. 10.1056/NEJMoa0908492 19843557

[B95] Roberts M. R. Qin L. Zhang D. Smith D. H. Tran A. C. Dull T. J. (1994). Targeting of human immunodeficiency virus-infected cells by CD8^+^ T lymphocytes armed with universal T-cell receptors. *Blood* 84 2878–2889. 7949163

[B96] Rossi J. J. June C. H. Kohn D. B. (2007). Genetic therapies against HIV. *Nat. Biotechnol.* 25 1444–1454. 10.1038/nbt1367 18066041 PMC4539027

[B97] Sagar M. Kirkegaard E. Long E. M. Celum C. Buchbinder S. Daar E. S. (2004). Human immunodeficiency virus type 1 (HIV-1) diversity at time of infection is not restricted to certain risk groups or specific HIV-1 subtypes. *J. Virol.* 78 7279–7283. 10.1128/JVI.78.13.7279-7283.2004 15194805 PMC421693

[B98] Samson M. Libert F. Doranz B. J. Rucker J. Liesnard C. Farber C. M. (1996). Resistance to HIV-1 infection in caucasian individuals bearing mutant alleles of the CCR-5 chemokine receptor gene. *Nature* 382 722–725. 10.1038/382722a0 8751444

[B99] Sattentau Q. J. Moore J. P. (1991). Conformational changes induced in the human immunodeficiency virus envelope glycoprotein by soluble CD4 binding. *J. Exp. Med.* 174 407–415. 10.1084/jem.174.2.407 1713252 PMC2118908

[B100] Schiffner T. Sattentau Q. J. Duncan C. J. (2013). Cell-to-cell spread of HIV-1 and evasion of neutralizing antibodies. *Vaccine* 31 5789–5797. 10.1016/j.vaccine.2013.10.020 24140477

[B101] Schumann K. Lin S. Boyer E. Simeonov D. R. Subramaniam M. Gate R. E. (2015). Generation of knock-in primary human T cells using Cas9 ribonucleoproteins. *Proc. Natl. Acad. Sci. U.S.A.* 112 10437–10442. 10.1073/pnas.1512503112 26216948 PMC4547290

[B102] Sengupta S. Siliciano R. F. (2018). Targeting the latent reservoir for HIV-1. *Immunity* 48 872–895. 10.1016/j.immuni.2018.04.030 29768175 PMC6196732

[B103] Shi B. Li J. Shi X. Jia W. Wen Y. Hu X. (2017). TALEN-mediated knockout of CCR5 confers protection against infection of human immunodeficiency virus. *J. Acquir. Immune Defic. Syndr.* 74 229–241. 10.1097/QAI.0000000000001190 27749600

[B104] Shimizu S. Hong P. Arumugam B. Pokomo L. Boyer J. Koizumi N. (2010). A highly efficient short hairpin RNA potently down-regulates CCR5 expression in systemic lymphoid organs in the hu-BLT mouse model. *Blood* 115 1534–1544. 10.1182/blood-2009-04-215855 20018916 PMC2830759

[B105] Slaymaker I. M. Gao L. Zetsche B. Scott D. A. Yan W. X. Zhang F. (2016). Rationally engineered Cas9 nucleases with improved specificity. *Science* 351 84–88. 10.1126/science.aad5227 26628643 PMC4714946

[B106] Stone D. Kiem H. P. Jerome K. R. (2013). Targeted gene disruption to cure HIV. *Curr. Opin. HIV AIDS* 8 217–223. 10.1097/COH.0b013e32835f736c 23478911 PMC4226633

[B107] Tebas P. Stein D. Tang W. W. Frank I. Wang S. Q. Lee G. (2014). Gene editing of CCR5 in autologous CD4 T cells of persons infected with HIV. *N. Engl. J. Med.* 370 901–910. 10.1056/NEJMoa1300662 24597865 PMC4084652

[B108] Thakkar N. Pirrone V. Passic S. Zhu W. Kholodovych V. Welsh W. (2009). Specific interactions between the viral coreceptor CXCR4 and the biguanide-based compound NB325 mediate inhibition of human immunodeficiency virus type 1 infection. *Antimicrob. Agents Chemother.* 53 631–638. 10.1128/AAC.00866-08 19047650 PMC2630669

[B109] Tsai S. Q. Nguyen N. T. Malagon-Lopez J. Topkar V. V. Aryee M. J. Joung J. K. (2017). CIRCLE-seq: a highly sensitive in vitro screen for genome-wide CRISPR-Cas9 nuclease off-targets. *Nat. Methods* 14 607–614. 10.1038/nmeth.4278 28459458 PMC5924695

[B110] Tsai S. Q. Zheng Z. Nguyen N. T. Liebers M. Topkar V. V. Thapar V. (2015). GUIDE-seq enables genome-wide profiling of off-target cleavage by CRISPR-Cas nucleases. *Nat. Biotechnol.* 33 187–197. 10.1038/nbt.3117 25513782 PMC4320685

[B111] Tycko J. Myer V. E. Hsu P. D. (2016). Methods for optimizing CRISPR-Cas9 genome editing specificity. *Mol. Cell* 63 355–370. 10.1016/j.molcel.2016.07.004 27494557 PMC4976696

[B112] Urnov F. D. Miller J. C. Lee Y. L. Beausejour C. M. Rock J. M. Augustus S. (2005). Highly efficient endogenous human gene correction using designed zinc-finger nucleases. *Nature* 435 646–651. 10.1038/nature03556 15806097

[B113] Vakulskas C. A. Dever D. P. Rettig G. R. Turk R. Jacobi A. M. Collingwood M. A. (2018). A high-fidelity Cas9 mutant delivered as a ribonucleoprotein complex enables efficient gene editing in human hematopoietic stem and progenitor cells. *Nat. Med.* 24 1216–1224. 10.1038/s41591-018-0137-0 30082871 PMC6107069

[B114] Van Lint C. Bouchat S. Marcello A. (2013). HIV-1 transcription and latency: an update. *Retrovirology* 10:67. 10.1186/1742-4690-10-67 23803414 PMC3699421

[B115] Verheyen J. Thielen A. Lubke N. Dirks M. Widera M. Dittmer U. (2018). Rapid rebound of a preexisting CXCR4-tropic HIV variant after allogeneic transplantation with CCR5 delta32 homozygous stem cells. *Clin. Infect. Dis.* 10.1093/cid/ciy565 [Epub ahead of print]. 30020413

[B116] Walker R. E. Bechtel C. M. Natarajan V. Baseler M. Hege K. M. Metcalf J. A. (2000). Long-term in vivo survival of receptor-modified syngeneic T cells in patients with human immunodeficiency virus infection. *Blood* 96 467–474. 10887107

[B117] Wang Q. Chen S. Xiao Q. Liu Z. Liu S. Hou P. (2017). Genome modification of CXCR4 by *Staphylococcus aureus* Cas9 renders cells resistance to HIV-1 infection. *Retrovirology* 14:51. 10.1186/s12977-017-0375-0 29141633 PMC5688617

[B118] Wang W. Ye C. Liu J. Zhang D. Kimata J. T. Zhou P. (2014). CCR5 gene disruption via lentiviral vectors expressing Cas9 and single guided RNA renders cells resistant to HIV-1 infection. *PLoS One* 9:e115987. 10.1371/journal.pone.0115987 25541967 PMC4277423

[B119] Weissenhorn W. Dessen A. Harrison S. C. Skehel J. J. Wiley D. C. (1997). Atomic structure of the ectodomain from HIV-1 gp41. *Nature* 387 426–430. 10.1038/387426a0 9163431

[B120] Wilen C. B. Wang J. Tilton J. C. Miller J. C. Kim K. A. Rebar E. J. (2011). Engineering HIV-resistant human CD4^+^ T cells with CXCR4-specific zinc-finger nucleases. *PLoS Pathog.* 7:e1002020. 10.1371/journal.ppat.1002020 21533216 PMC3077364

[B121] Wilkin T. J. Su Z. Kuritzkes D. R. Hughes M. Flexner C. Gross R. (2007). HIV type 1 chemokine coreceptor use among antiretroviral-experienced patients screened for a clinical trial of a CCR5 inhibitor: AIDS Clinical Trial Group A5211. *Clin. Infect. Dis.* 44 591–595. 10.1086/511035 17243065

[B122] Wolfs T. F. Zwart G. Bakker M. Goudsmit J. (1992). HIV-1 genomic RNA diversification following sexual and parenteral virus transmission. *Virology* 189 103–110. 10.1016/0042-6822(92)90685-I 1376536

[B123] Wolinsky S. M. Wike C. M. Korber B. T. Hutto C. Parks W. P. Rosenblum L. L. (1992). Selective transmission of human immunodeficiency virus type-1 variants from mothers to infants. *Science* 255 1134–1137. 10.1126/science.1546316 1546316

[B124] Xu K. Acharya P. Kong R. Cheng C. Chuang G. Y. Liu K. (2018). Epitope-based vaccine design yields fusion peptide-directed antibodies that neutralize diverse strains of HIV-1. *Nat. Med.* 24 857–867. 10.1038/s41591-018-0042-6 29867235 PMC6358635

[B125] Xu L. Yang H. Gao Y. Chen Z. Xie L. Liu Y. (2017). CRISPR/Cas9-Mediated CCR5 ablation in human hematopoietic stem/progenitor cells confers HIV-1 resistance in vivo. *Mol. Ther.* 25 1782–1789. 10.1016/j.ymthe.2017.04.027 28527722 PMC5542791

[B126] Yang O. O. Tran A. C. Kalams S. A. Johnson R. P. Roberts M. R. Walker B. D. (1997). Lysis of HIV-1-infected cells and inhibition of viral replication by universal receptor T cells. *Proc. Natl. Acad. Sci. U.S.A.* 94 11478–11483. 10.1073/pnas.94.21.11478 9326635 PMC23511

[B127] Ye L. Wang J. Beyer A. I. Teque F. Cradick T. J. Qi Z. (2014). Seamless modification of wild-type induced pluripotent stem cells to the natural CCR5Delta32 mutation confers resistance to HIV infection. *Proc. Natl. Acad. Sci. U.S.A.* 111 9591–9596. 10.1073/pnas.1407473111 24927590 PMC4084478

[B128] Yi G. Choi J. G. Bharaj P. Abraham S. Dang Y. Kafri T. (2014). CCR5 gene editing of resting CD4^+^ T cells by transient ZFN expression from HIV envelope pseudotyped nonintegrating lentivirus confers HIV-1 resistance in humanized mice. *Mol. Ther. Nucleic Acids* 3:e198. 10.1038/mtna.2014.52 25268698 PMC4222653

[B129] Yu A. Q. Ding Y. Lu Z. Y. Hao Y. Z. Teng Z. P. Yan S. R. (2018). TALENs-mediated homozygous CCR5Delta32 mutations endow CD4^+^ U87 cells with resistance against HIV1 infection. *Mol. Med. Rep.* 17 243–249. 10.3892/mmr.2017.7889 29115572 PMC5780131

[B130] Yu S. Yao Y. Xiao H. Li J. Liu Q. Yang Y. (2018). Simultaneous knockout of CXCR4 and CCR5 genes in CD4^+^ T cells via CRISPR/Cas9 confers resistance to both X4- and R5-tropic human immunodeficiency virus type 1 infection. *Hum. Gene Ther.* 29 51–67. 10.1089/hum.2017.032 28599597

[B131] Yuan J. Wang J. Crain K. Fearns C. Kim K. A. Hua K. L. (2012). Zinc-finger nuclease editing of human cxcr4 promotes HIV-1 CD4^+^ T cell resistance and enrichment. *Mol. Ther.* 20 849–859. 10.1038/mt.2011.310 22273578 PMC3321595

[B132] Yukl S. A. Boritz E. Busch M. Bentsen C. Chun T. W. Douek D. (2013). Challenges in detecting HIV persistence during potentially curative interventions: a study of the Berlin patient. *PLoS Pathog.* 9:e1003347. 10.1371/journal.ppat.1003347 23671416 PMC3649997

[B133] Zhu C. Gupta A. Hall V. L. Rayla A. L. Christensen R. G. Dake B. (2013). Using defined finger-finger interfaces as units of assembly for constructing zinc-finger nucleases. *Nucleic Acids Res.* 41 2455–2465. 10.1093/nar/gks1357 23303772 PMC3575815

[B134] Zhu T. Mo H. Wang N. Nam D. S. Cao Y. Koup R. A. (1993). Genotypic and phenotypic characterization of HIV-1 patients with primary infection. *Science* 261 1179–1181. 10.1126/science.8356453 8356453

[B135] Zou Y. R. Kottmann A. H. Kuroda M. Taniuchi I. Littman D. R. (1998). Function of the chemokine receptor CXCR4 in haematopoiesis and in cerebellar development. *Nature* 393 595–599. 10.1038/31269 9634238

